# A single neonatal administration of Bisphenol A induces higher tumour weight associated to changes in tumour microenvironment in the adulthood

**DOI:** 10.1038/s41598-017-10135-1

**Published:** 2017-09-05

**Authors:** Margarita Isabel Palacios-Arreola, Karen Elizabeth Nava-Castro, Víctor Hugo Del Río-Araiza, Nashla Yazmín Pérez-Sánchez, Jorge Morales-Montor

**Affiliations:** 1 Departamento de Inmunología, Instituto de Investigaciones Biomédicas, Universidad Nacional Autónoma de México, AP 70228, Ciudad de Mexico, CP 04510 Mexico; 2 Laboratorio de Genotoxicología y Mutagénesis Ambientales, Departamento de Ciencias Ambientales, Centro de Ciencias de la Atmósfera, Universidad Nacional Autónoma de Mexico, CP 04510 Ciudad de Mexico, Mexico

## Abstract

BPA is an oestrogenic endocrine disrupting chemical compound. Exposure to BPA in as early as pregnancy leads to lifelong effects. Since endocrine and immune systems interact in a bidirectional manner, endocrine disruption may cause permanent alterations of the immune system, affecting a future anti-tumoral response. Neonate (PND 3) female syngeneic BALB/c mice were exposed to a single dose of 250 µg/kg BPA. Once sexual maturity was reached, a mammary tumour was induced injecting 4T1 cells *in situ*, these cells are derived from a spontaneous adenocarcinoma in a BALB/c mouse and therefore allows for an immunocompetent recipient. After 25 days of injection, showing no major endocrine alterations, BPA-exposed mice developed larger tumours. Tumour leukocytic infiltrate analysis revealed a higher proportion of regulatory T lymphocytes in the BPA-exposed group. RT-PCR analysis of tumour samples showed a decreased expression of TNF-α and IFN-γ, as well as the M2 macrophage marker Fizz-1 in the BPA-exposed group. Flow cytometry analysis revealed differences in ERα expression by T lymphocytes, macrophages and NK cells, both associated to BPA exposure and tumour development. These findings show a new aspect whereby early life BPA exposure can contribute to breast cancer development and progression by modulating the anti-tumoral immune response.

## Introduction

Human activity has led to an increased amount of environmental contaminants, including endocrine disrupting chemicals (EDCs). EDCs are defined as exogenous compounds interfering with the synthesis, secretion, metabolism, transport, mechanism of action, and elimination of endogenous steroid hormones^1^. Bisphenol A (BPA), an oestrogenic EDC, is characterized by its high affinity to nuclear oestrogen receptors (ERα and ERβ)^2, 3^. Moreover, BPA is widely used in the production of polycarbonate plastics, epoxy resins (used in food containers and can linings, respectively), as well as dental sealants; all of them used daily by people. BPA can leak from these materials when exposed to high temperatures, acidic conditions, or saliva, thus leading to human exposure^4–6^.

BPA causes several endocrine alterations, not only in the reproductive organs^7–11^ but also in mammary gland development^12–17^, and brain sexual differentiation^18–20^. The nature and magnitude of these effects depends on the dose, exposure course, and the developmental stage at which the exposure occurs. Regarding the latter, endocrine disruption during critical developmental stages could lead to lifelong effects.

It is well known that BPA exposure can occur as early as during gestation, as evidenced by reports of BPA presence in amniotic fluid, foetal serum, and breast milk^21, 22^. In this regard, there is an existing concern about the effects that BPA could exert in a developing organism, including the immune system.

Concerning the immune system in mice, the critical developmental stages encompass both the gestational and neonatal periods. Beyond the immune organ formation and hematopoiesis, the maturation of the immune system occurs between birth and ﻿post natal day 30(PND 30﻿)^23^. Moreover, it has been demonstrated that PND 2–4 are critical for T lymphocyte development, since thymectomy or cyclosporine treatment at this age, but not later, results in multi-organ autoimmunity^24–26^.

On the other hand, cancer is the second cause of death worldwide; according to the WHO^27^, it accounted for 8.8 million deaths in 2015. Breast cancer is among the most prevalent types of cancer, and several environmental factors have been linked to an increased incidence of this ailment. When talking about cancer, there are multiple factors that should be taken into account, not only those contributing to cellular stress, DNA damage, and emergence of transformed cells, but also the mechanisms responsible for the detection and elimination of cancer cells. In this context, the immune system, particularly cell populations such as NK cells, T lymphocytes, regulatory T lymphocytes (Treg), and Tumour Associated Macrophages (TAMs), as well as their secreted cytokines, play a major role in preventing cancer development or controlling its progression^28, 29^.

The endocrine and immune systems are not independent physiological entities, but parts of a net of bidirectional interactions mediated by hormones, cytokines and even neurotransmitters which have been widely documented in recent years^30, 31^. Regarding sexual steroid hormones, they regulate the distribution and functionality of several immune cell populations, modulating the immune response, maturation and selection of thymocytes, lymphoid proliferation, and cytokine production^31^. The modulatory effects of sex steroids are mediated by specific receptors present in immune cells, namely oestrogen receptors (ERα, ERβ), progesterone receptor (PR), and androgen receptor (AR), as well as membrane oestrogen and progesterone receptors, among others^31^.

Since sexual steroids modulate the immune system, exogenous agents such as EDCs could also influence it.

Therefore, we decided to assess the potential alteration of the anti-tumoral immune response caused by BPA exposure at a critical developmental stage, specifically during the neonatal period. Our results clearly demonstrate that BPA administered during the neonatal period has an impact on tumour size, weight, and immune-endocrine microenvironment during adult life.

## Results

### Endocrine Parameters

The experimental strategy involves the development of a tumour within hormone-sensitive tissue; therefore, it was important to discriminate whether the observed effects could be attributed to the direct or indirect effect of neonatal endocrine disruption on the immune system or caused by a persistent hormonal alteration.

**Figure 1:**
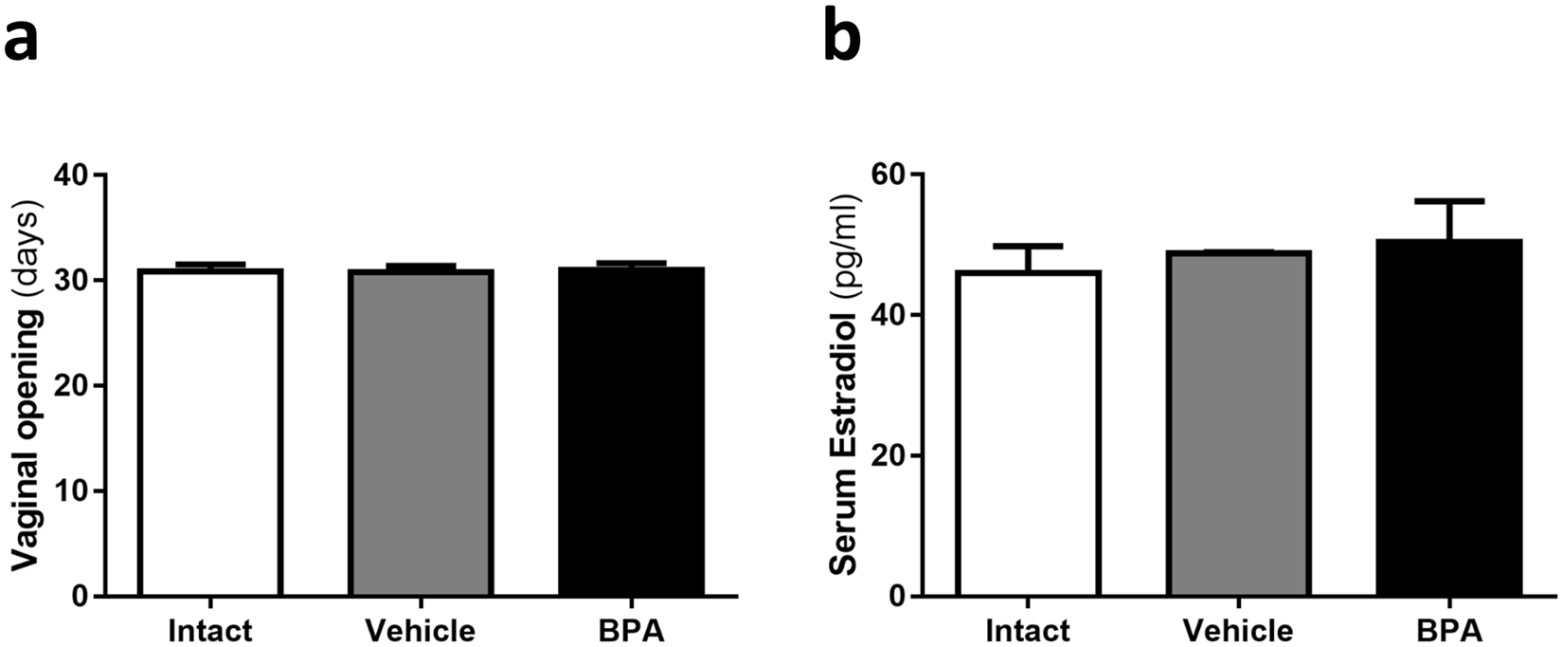
Endocrine parameters assessment. (**a**) Vaginal opening as puberty onset indicator. (**b**) Serum 17β-Oestradiol levels at diestrus phase at 12 weeks of age measured by EIA. Data from 2 independent experiments are expressed as mean ± SD; n = 14 in (**a**) and 8 in (**b**).

In order to assess the potential reproductive effects of a single 250 μg/kg bw BPA dose, puberty onset was determined by the age of vaginal opening. Contrary to what has been reported with higher or more prolonged exposures, neonatal BPA exposure did not alter puberty onset (Fig. 1a)^8, 9^.

In addition, the oestrous cycle was also monitored once sexual maturity was fully established (8 weeks old), finding no difference between the experimental groups (data not shown). Furthermore, BPA exposure had no effect on baseline levels of serum oestradiol during the diestrus phase (Fig. 1b).

### Tumour size and weight

**Figure 2:**
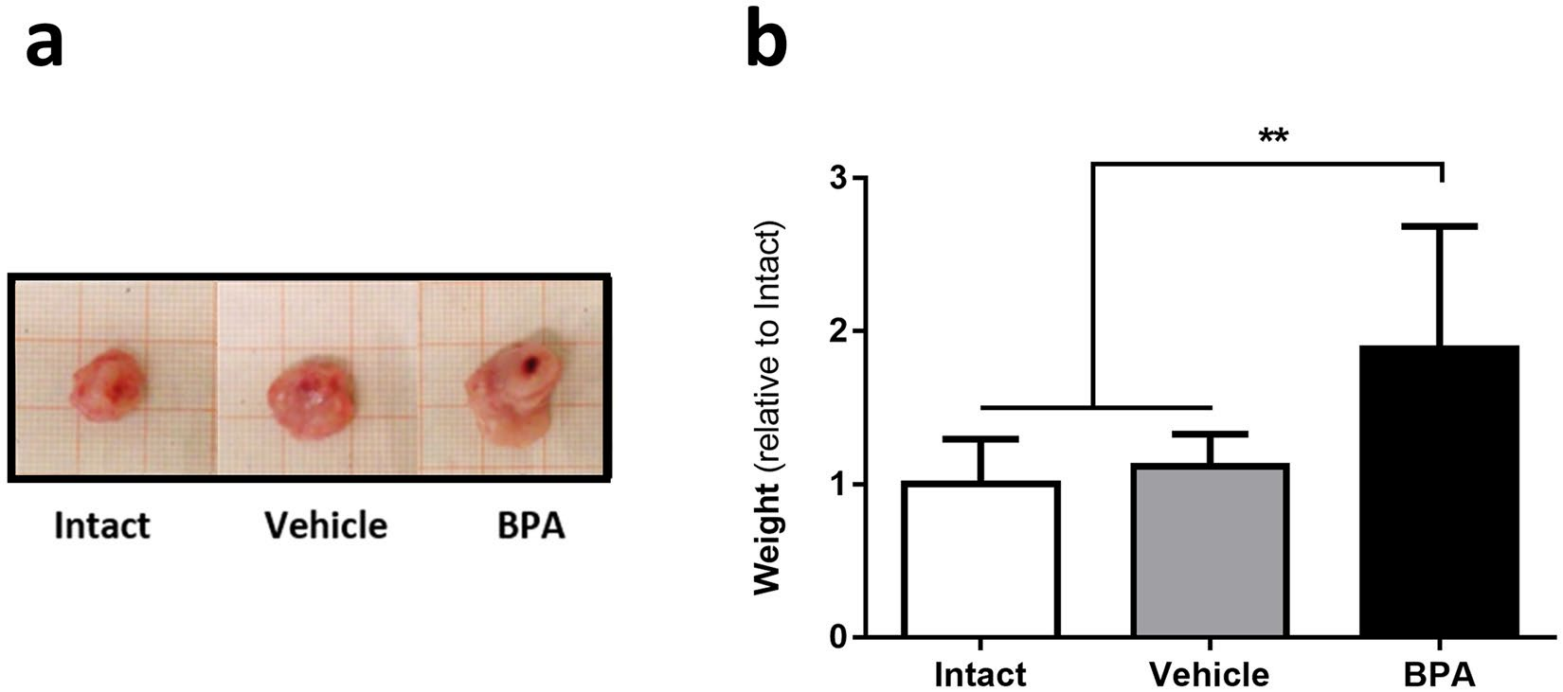
Tumour growth. (**a**) Representative images of tumours of the three experimental groups; millimetric grid as background. (**b**) Evaluation of final weight after 25 days of tumour development; data from 3 independent experiments are expressed as mean ± SD; n = 12; **p = 0.007 vs Intact, *p* = 0.0025 vs Vehicle.

A significant promotion of tumour development was observed in the BPA-exposed group. At 25 days after the inoculation of tumour cells, it was evident that the mice subjected to neonatal BPA exposure developed larger tumours (Fig. 2a). Indeed, after measuring tumour weight, those found in BPA-exposed mice showed an 88% increase in weight when compared to unexposed groups (Fig. 2b), *i.e*. in the BPA group, the mean relative weight was 1.88 (CI 1.48, 2.28) compared to a mean relative weight of 1 (CI 0.8, 1.19) for the Intact group and a mean relative weight of 1.12 (CI 0.98, 1.25) for the Vehicle group.

### Tumour infiltrate

**Figure 3:**
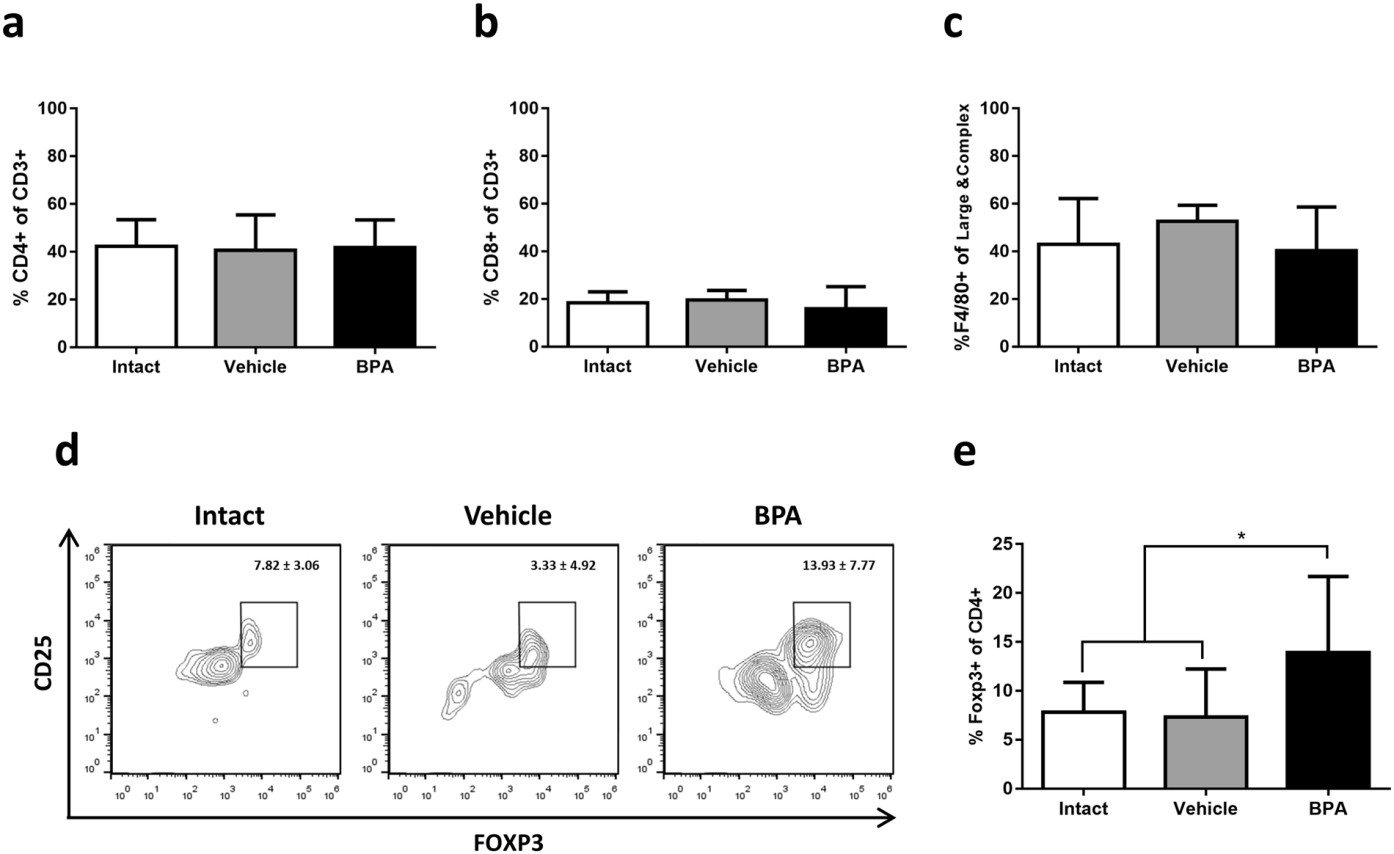
Analysis of tumour leucocytic infiltrate. Determination of immune subpopulations by flow cytometry, (**a**) T helper lymphocytes, (**b**) cytotoxic T lymphocytes, (**c**) macrophages and (**e**) Treg; *p = 0.0335 vs Intact, *p* = 0.0247 vs Vehicle. Data from 3 independent experiments are expressed as mean ± SD; n = 12. (**d**) Representative contour representations of cytometric analysis of Treg percentage; gate: CD3^+^, CD4^+^ from 10,000 lymphocytes collected.

The flow cytometry analysis of leukocytic infiltrate in tumours revealed that the cellular composition was mostly similar between the groups (Fig. 3a–c); however, Treg were increased in the BPA-exposed group (Fig. 3d,e), comprising a 13.93% (CI 10.06, 17.79) of CD4+ lymphocytes when compared to 7.82% (CI 5.77, 9.87) and 7.33% (CI 3.81, 10.85) in intact and vehicle groups, respectively.

**Figure 4:**
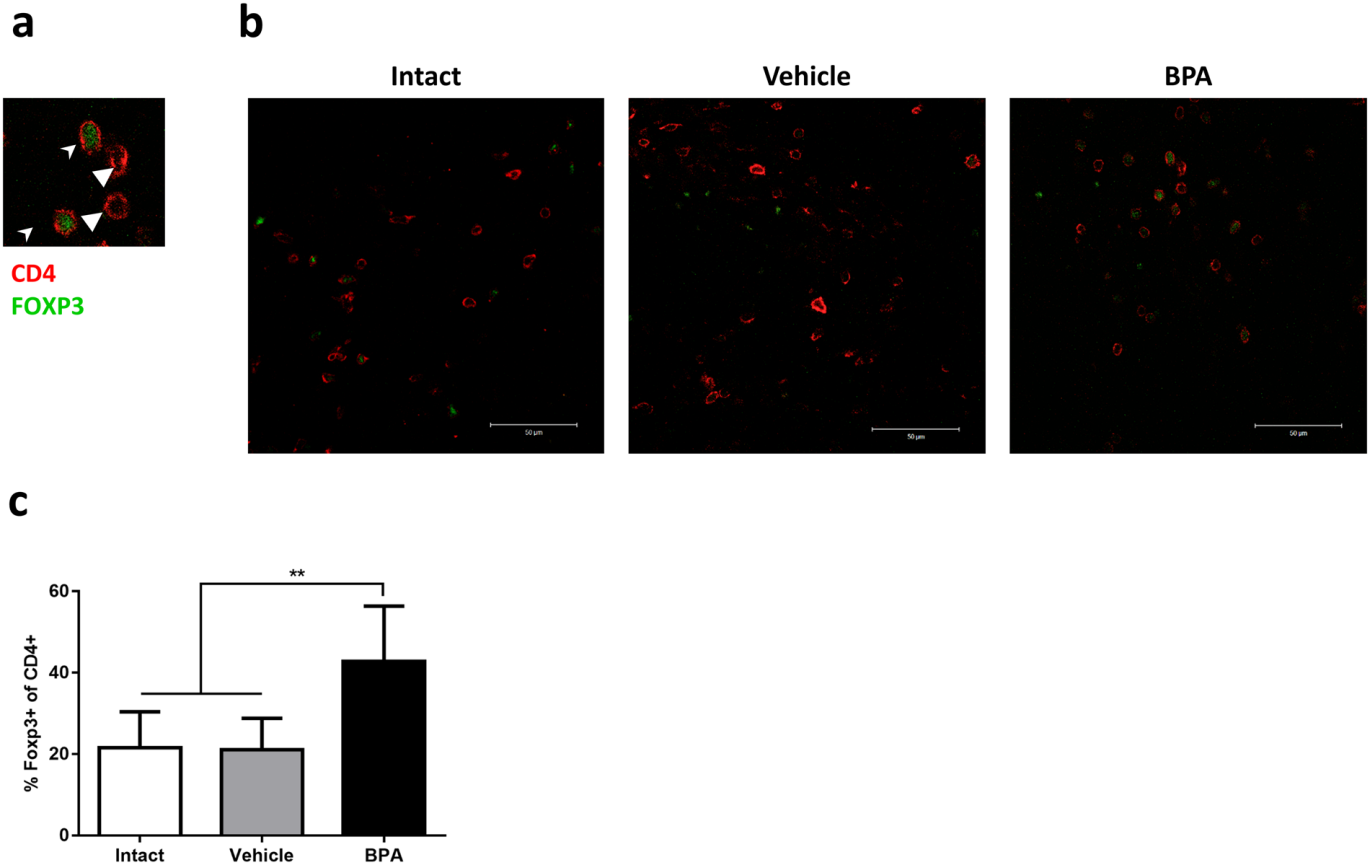
Evaluation of tumour Treg abundance by immunofluorescence. (**a**) Representative identification of T helper lymphocytes (white triangles △) and Treg (white arrowheads  ) by immunofluorescence staining of membranal CD4 (red) and nuclear Foxp3 (green); scale bar, 50 μm; original magnification, ×40. (**b**) Representative images of immunofluorescence staining of tumour samples. (**c**) Treg proportion expressed as the percentage of Foxp3^+^ cells of CD4^+^ cells; **p = 0.0031 vs Intact, *p* = 0.0070 vs Vehicle; data from 2 independent experiments are expressed as mean ± SD; n = 8.

Further, immunofluorescence analysis of tumour sections confirmed the greater proportion of regulatory T lymphocytes within the tumours of BPA-exposed mice (Fig. 4), comprised by an average of 42.8% (CI 30.27, 55.33) of CD4 + cells, compared to 21.54% (CI 14.14, 28.94) in the intact group and 21.88% (CI 11.52, 30.64) in the vehicle group.

**Figure 5:**
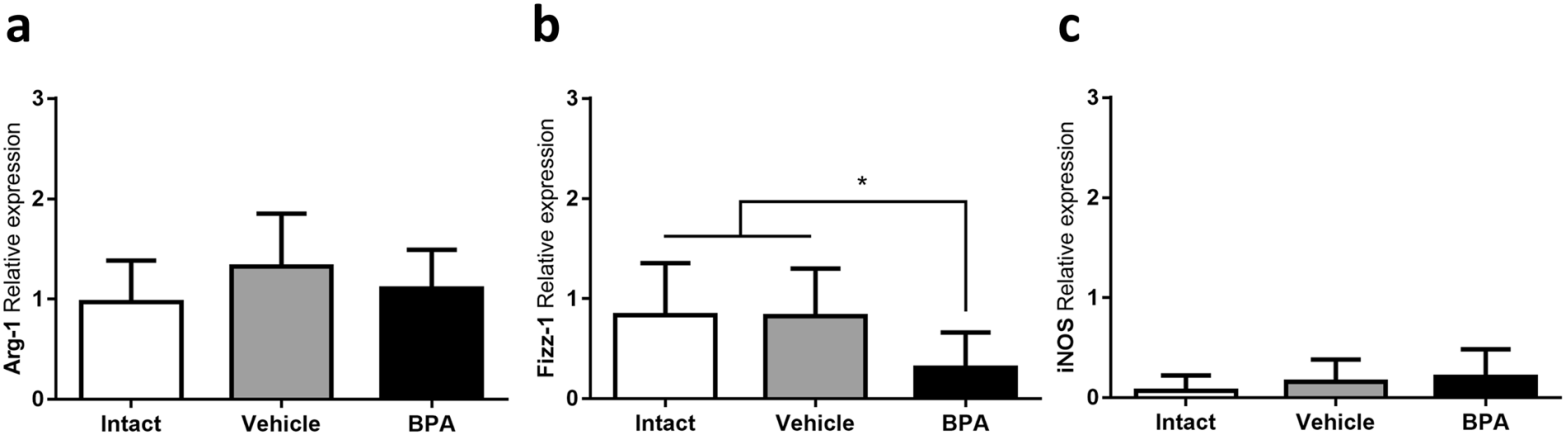
TAMs activation profile. Relative expression of alternative activation markers (**a**) Arginase and (**b**) Fizz-1 and classical activation marker (**c**) iNOs determined by RT-PCR. Expression relative to 18 S ribosomal subunit as constitutive expression reference. *p = 0.0326 vs Intact, *p* = 0.0476 vs Vehicle; data from 2 independent experiments are expressed as mean ± SD; n = 10.

Tumour associated macrophages (TAMs) represent an important subset of leukocytic infiltrate in many kinds of tumours, where they may display a classical (M1) or an alternative (M2) activation phenotype. By means of RT-PCR, the relative expression of classical (iNOS) and alternative (Arginase, Fizz-1 and Ym-1) activation markers was assessed. TAMs of all groups were clearly activated in an alternative manner, as evidenced by a higher arginase expression when compared to iNOS (Fig. 5a,c). However, TAMs from the BPA-exposed group showed a lower Fizz-1 relative expression mean (0.307; CI 0.09, 0.51) when compared to the intact (0.834; CI 0.52, 1.27) and vehicle groups (0.821; CI 0.47, 1.26) (Fig. 5b). Ym-1, on the other hand, was not expressed in any of the tumour samples.

In contrast to the tumour infiltrate, which usually shows Treg enrichment, the spleen of tumour-bearing mice appeared nearly devoid of these cells. Further, a major finding involved the virtual absence of NK cells from the tumour infiltrate and were drastically diminished in the periphery of all tumour-bearing mice, in contrast to healthy ones.

### Intra-tumoral cytokine expression

**Figure 6:**
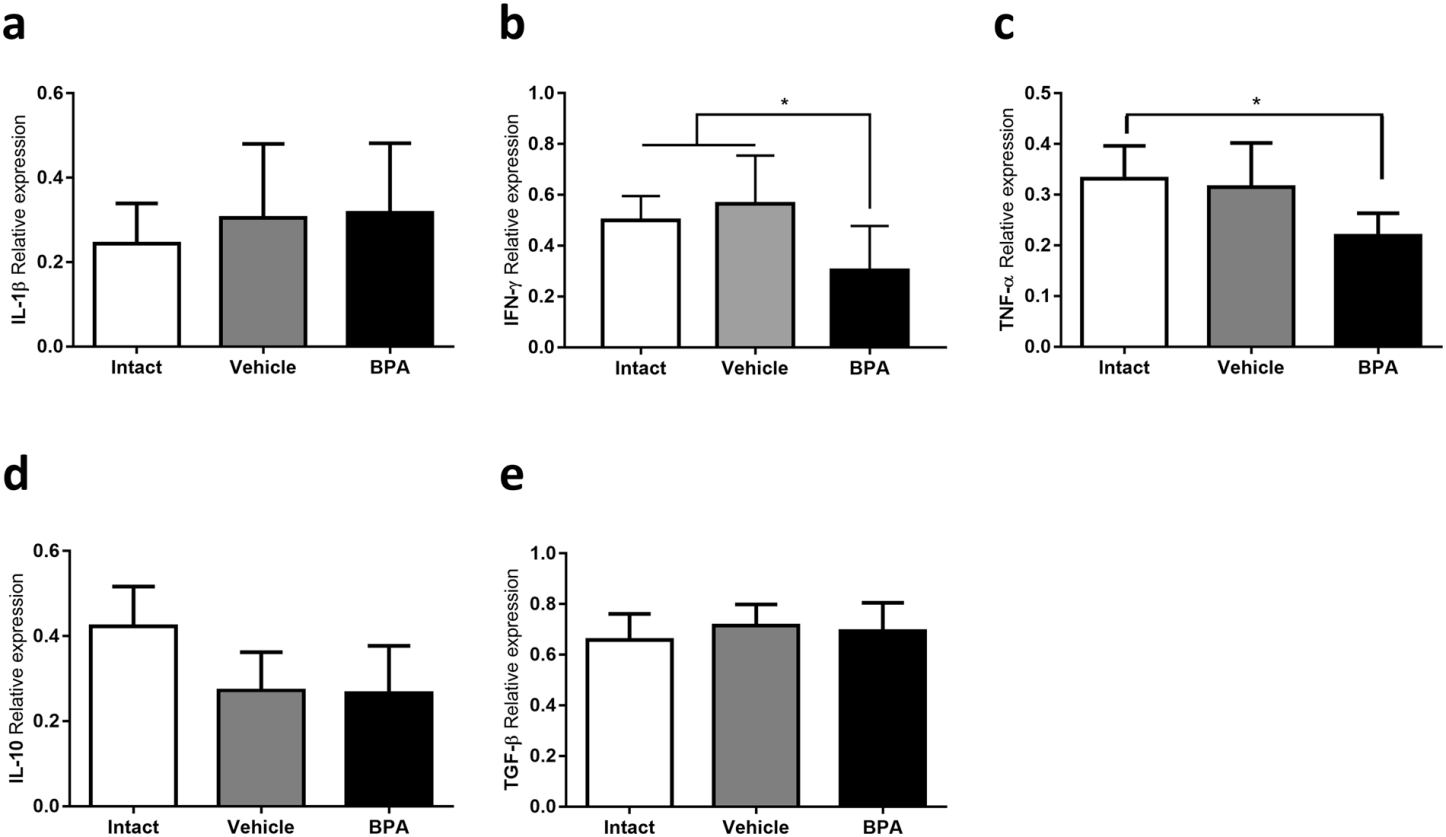
Intratumoural cytokine expression. Relative expression of proinflammatory (**a**) IL-1β, (**b**) IFN-γ, (**c**) TNF-α, and immunomodulatory cytokines (**d**) IL-10 and (**e**) TGF-β, determined by RT-PCR. Expression relative to 18 S ribosomal subunit as constitutive expression reference. In (**b**) *p = 0.0216 vs Intact, *p* = 0.0034 vs Vehicle; in (**c**) *p = 0.0244 vs Intact; data from 2 independent experiments are expressed as mean ± SD; n = 10.

As part of the tumour immunological microenvironment analysis, we determined the relative expression of pro-inflammatory (IL-1β, TNF-α, and IFN-γ), as well as regulatory (IL-10 and TGF-β) cytokines in tumour tissue. The cytokine milieu in all groups is clearly skewed towards a regulatory phenotype, as denoted by the predominance of TGF-β relative expression (Fig. 6e).

However, it drew our attention that, whilst having a greater regulatory T proportion, the tumour samples of the BPA-exposed group did not exhibit a greater expression of TGF-β, nor IL-10 (Fig. 6d,e). Moreover, both TNF-α and IFN-γ expression was decreased in the BPA-exposed group (Fig. 6b,c), suggesting that the overall tumour microenvironment in this group displays a lesser pro-inflammatory character.

### ER-α expression

The cells of the immune system are modulated by sex steroids, mediated by receptors present in these cells. Given the oestrogenic character of the endocrine disruption caused by BPA, we analysed ERα expression in the main immune subpopulations at both local and systemic levels.

**Figure 7:**
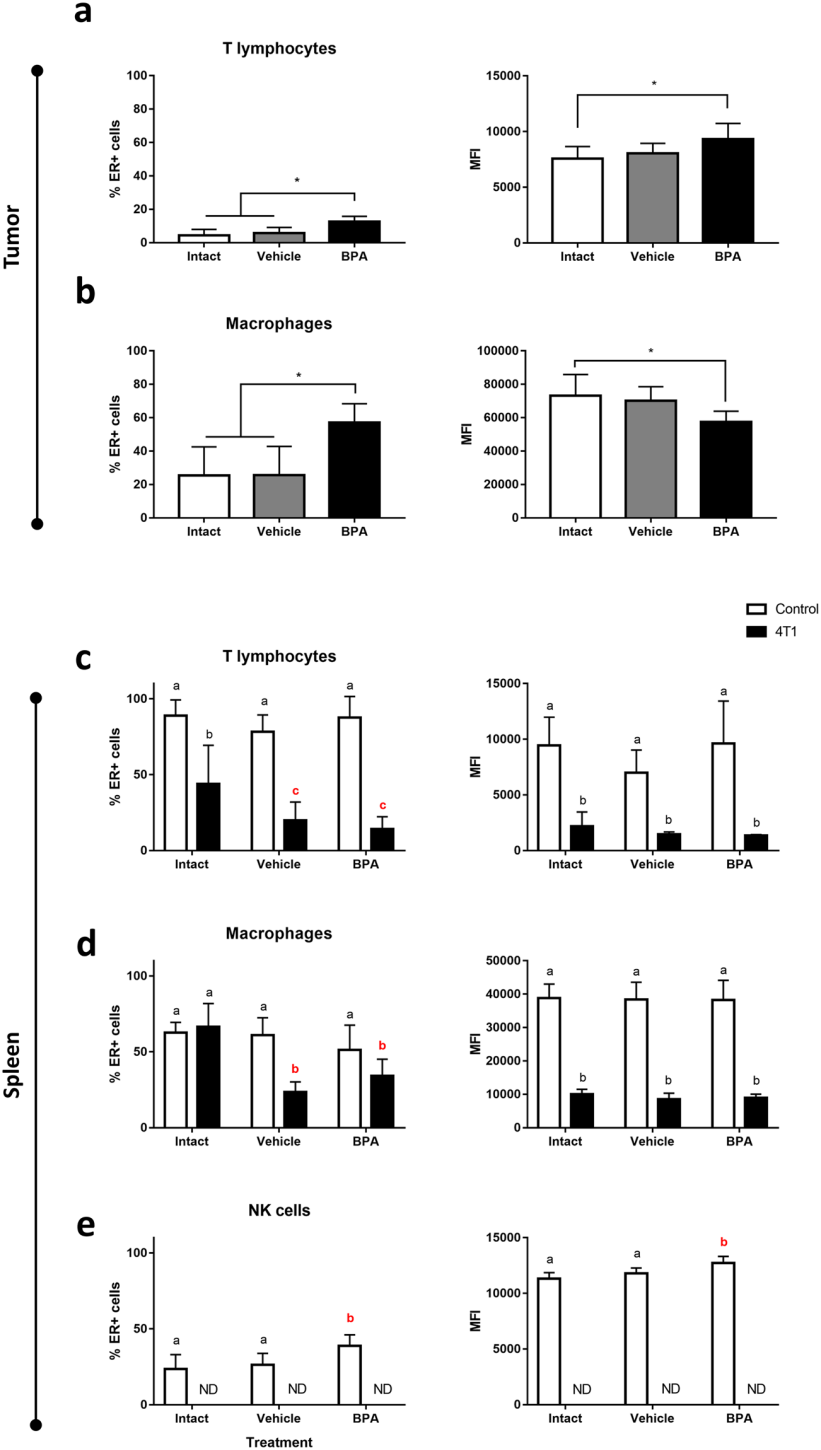
Flow cytometry evaluation of ERα expression in immune cells. Percentage of ERα positive cells (left panel) and ERα expression level (right panel) of immune cells in the tumour microenvironment (**a**,**b**) and in the spleen (**c**–**e**). (**a**,**c**) T lymphocytes, (**b**,**d**) macrophages, (**e**) NK cells. Data from 2 independent experiments are expressed as mean ± SD; n = 10. *p < 0.05 compared with unexposed controls. In (**c**–**e**) white bars, control = no tumour induction; black bars, 4T1 = tumour induction; letters above each column indicate statistical differences among groups: a, no significant difference; b, *p* < 0.05 compared to a; c, *p* < 0.05 compared to b; ND, no data available since this subpopulation was absent from the tissue.

We observed a greater proportion of ERα-positive T lymphocytes in the tumour microenvironment of BPA-exposed mice, accompanied by an elevated expression level of the receptor (Fig. 7a).

The macrophage infiltrate of the BPA-exposed group also showed a larger percentage of ERα-positive cells; however, ERα expression in this group was significantly lower (Fig. 7b). This is a noteworthy finding, as it could account for the decreased Fizz-1 expression found in the same tissue, considering that oestrogen signalling promotes the expression of this protein^32^.

At systemic level, we found that the T lymphocytes of tumour-bearing mice had lower ERα expression compared to their healthy counterparts (Fig. 7c). Though this phenomenon was observed in all groups, the deviation in ERα expression between healthy and tumour-bearing mice was greater in the Vehicle and BPA groups than in the Intact group. A similar behaviour was noted in splenic macrophages, where the proportion of ERα-positive cells was decreased in tumour-bearing mice of the Vehicle and BPA-exposed groups (Fig. 7d). As ERα expression remained constant in healthy mice regardless of the neonatal treatment, these findings suggest that neonatal stress, but not BPA exposure could play a role in the modulation of ERα expression in response to tumour development.

The analysis of ERα expression in NK cells was only possible at systemic level, since this population was absent from the tumour microenvironment at this time point. Moreover, the spleens of tumour-bearing mice was depleted of NK cells. Nevertheless, the analysis on ERα expression in NK cells of healthy mice revealed that neonatal BPA-exposure not only increased the percentage of ERα positive cells, but also increased its expression level (Fig. 7e).

## Discussion

Our experimental model involves the growth of a mammary adenocarcinoma tumour embedded within the mammary gland of a mouse^33^, resulting in a hormone-sensitive system. Originally, the use of 4T1 cells was contemplated as a strategy excluding the use of oestradiol as a growth factor, given that this cell line does not express the oestrogen receptor ERα^34–36^; however, we detected the presence of this receptor in the 4T1 cell line. This posed the possibility that the observed changes in tumour growth could be attributed to either endocrine (*e.g*. higher serum oestradiol levels) or immunological factors, or both. Whilst it has been well documented that BPA administered in rodents exerts several effects on the endocrine system, particularly on the reproductive axis, such as early puberty onset, oestrous cycle alterations, and even altered development of the mammary gland^8, 9, 12, 37^, the majority of these effects occur after a prolonged perinatal or pre-pubertal exposure to higher doses^38^.

Therefore, we evaluated the potential endocrine alterations produced by the tested exposure scheme, *i.e*. a single dose of 250 µg/kg bw at postnatal day 3. As a result, we did not observed any alterations regarding puberty onset, regularity of the oestrous cycle, or basal oestradiol levels in serum. The latter suggests that the observed effects, regarding tumour progression, are mainly due to immunological factors.

Remarkably, tumour growth was promoted as a result of a single neonatal BPA exposure. Previous studies have associated BPA with breast cancer risk, mainly as a consequence of mammary gland alterations and carcinogenesis susceptibility^14–17, 39, 40^. Nevertheless, our results show a new aspect whereby BPA can contribute to breast cancer development and progression by modifying the anti-tumoral immune response.

The analysis of the tumour leukocytic infiltrate showed that the BPA-exposed group had a larger proportion of Treg lymphocytes, in accordance with a greater tumour growth. It is known that Tregs express the immunomodulatory cytokines IL-10 and TGF-β while at the same time limiting the production of pro-inflammatory cytokines by other cells^41–43^. It appeared odd that we did not observe any changes in the expression of immunomodulatory cytokines in the BPA group; instead, this group showed a lower expression of the pro-inflammatory cytokines IFN-γ and TNF-α; therefore, the tumour microenvironment of this group was indeed modified in general terms. In any case, the expression level was assessed at mRNA level, which is limiting when considering the post-transcriptional regulations that may be affecting final protein synthesis. Regardless, the immunomodulatory role of Treg lymphocytes is complex, as their effector mechanisms are not limited to the cytokine expression, and also mediate contact-dependent immunosuppression and IL-2 depletion.

Moreover, tumour microenvironment is not only defined by adaptive immunity cells; in fact, TAMs comprise a very important population. It is known that TAMs acquire a particular phenotype while in the tumour microenvironment, more similar to alternative (M2) than classical (M1) activation^44–46^. TAMS found in the analysed tumour samples did show a greater expression of arginase (M2 marker) compared to iNOS (M1 marker); however, it results interesting that the samples from BPA-exposed mice showed lower Fizz-1 expression. Although Fizz-1 is considered a M2 marker, the role of this protein is still not clear, and while it has been postulated that it can act as a modulator of type 2 inflammation in the lungs, there is no information concerning its role in the context of tumour microenvironment.

One of the most relevant aspects of this study was the evaluation of the major oestrogen receptor in immune cells (ERα). As previously stated, sexual steroid hormones modulate the response of immune cells *via* its receptors; the effects that these hormones exert are diverse and greatly dependent on cell type, hormone concentration and combination, and even the expressed isoforms of the receptors^47–49^. Even in the context of an invariable hormone concentration, a change in the expression of a receptor may modify the sensibility of a cell to this hormone; in other words, it is not only a matter of how much hormone is present, but also how much the cell is sensing.

Considering that immune cells express a plethora of hormonal receptors, it is not possible to draw definitive conclusions based only on the evaluation of ERα. Nevertheless, it is possible to associate the changes in the expression of this receptor in immune cells to the changes observed in the modulation of these cells. For example, it has been reported that oestrogen promotes Treg differentiation *via* ERα^50–52^, which could account in part for the increased Treg ratio in the tumour microenvironment, as T lymphocytes in this tissue showed a higher ERα expression level. Furthermore, oestrogen has also been shown to influence macrophage polarization, promoting an alternative activation during skin repair^32^. In this manner, the modulation of ERα in TAMs could also relate to the observed changes in their activation profile.

As for what caused the changes in ERα expression, it has been reported that BPA exposure during critical developmental stages is able to modify the epigenetic patterns, affecting the expression of oestrogen receptors in reproductive tissues and in the brain^20, 53, 54^. Considering the latter, it is a plausible possibility that immune cells are also subjected to epigenetic modulation due to developmental BPA exposure. However, such hypothesis needs to be confirmed.

## Conclusion

The present work demonstrates that exposure to a single dose of BPA during the neonatal period induces changes in the immune system, leading to a differential anti-tumoral immune response during adulthood, causing greater tumour growth. This disparity is characterized by a greater intra-tumoral Treg proportion, decreased expression of pro-inflammatory cytokines, and a slightly different TAM activation profile. Because BPA exposure modified the expression pattern of ERα in immune cells, it is a plausible mechanism underlying the altered immune response caused by BPA exposure.

## Methods

### Ethics statement

Animal care and experimental practice were conducted at the Unidad de Modelos Biológicos (UMB) in the Instituto de Investigaciones Biomédicas (IIB), Universidad Nacional Autónoma de México. All experimental procedures in the animals were approved by the Institutional Care and Animal Use Committee (CICUAL), permit number 155, adhering to Mexican regulation (NOM-062-ZOO-1999), and in accordance with the recommendations from the National Institute of Health (NIH) of the United States of America (Guide for the Care and Use of Laboratory Animals). Euthanasia of experimental animals was performed humanely by cervical dislocation after anaesthesia with 5% sevofluorane (Abbot, México).

### Animals

Mice of the syngeneic strain BALB/c AnN (H2-d) were purchased from Harlan México (Facultad de Química, UNAM, México). The animals were housed at UMB with controlled temperature (22 °C) and 12-hrs light-dark cycles, with water and Purina LabDiet 5015 (Purina, St. Louis MO) chow *ad libitum*. After neonatal treatment, only female mice were used for experimentation.

### Neonatal BPA exposure

To resemble the human final gestational stage and aiming at the murine critical T lymphocytes developmental window, mice were exposed at PND3.

Briefly, 72 hours after birth female pups were identified by ano-genital distance. Only female pups received treatment, though whole litters were assigned to experimental groups to avoid pup reallocation stress. The Intact group received no neonatal treatment. The vehicle group received a dorsal subcutaneous injection of 20 µl corn oil vehicle (Sigma, St. Louis MO). The BPA group received 250 µg/kg bw of BPA. Given that neonate rodents have minimal glucuronidation activity, which is the major metabolic mechanism for BPA clearance^55, 56^, this dose approximates to a brief, 5 day exposure to the FDA reference dose of 50 µg/kg bw/day, but performed in a single administration, thus avoiding excessive manipulation stress.

Though the main exposure route is commonly oral, subcutaneous injection was selected instead as no difference between oral and subcutaneous routes are observed in neonate mice in this case^57^.

Pups were weaned at 21 days of age and placed in standard cages, 5 mice per cage.

### Assessment of endocrine parameters

Vaginal opening. From 25 days old forth, the vaginal opening was examined by holding the mice in a dorsal restraint and using a light extension of the peri-vaginal skin.

Estrous cycle. At 8 weeks old, the oestrous cycle was assessed using a vaginal smear wash of 50 µl saline solution (PiSA, Guadalajara México), followed by Giemsa stain and light microscope observation.

Serum Oestradiol. Serum samples obtained at sacrifice, corresponding to the diestrus phase, were used to determine oestradiol levels, using EIA DetectX® Serum 17βOestradiol kit (Arbor Assays, Ann Arbor MI), according to manufacturer’s protocol.

### Cell culture

The 4T1 cell line (ATCC^®^ CRL-2539) was kindly donated by Dr. Pedro Ostoa-Saloma and cultivated in RMPI 1640 medium (Sigma, St. Louis MO) supplemented with 10% FBS (ByProductos, Guadalajara México). Subculture was performed at 70–80% confluency. After a second subculture, the cells were harvested and resuspended in 0.9% saline to a concentration of 250,000 cells/ml for inoculation.

### Mammary tumour induction

Upon sexual maturity (8 weeks old), mice of every exposure group were randomized into secondary experimental groups, *i.e*. Control (without tumour induction) and 4T1 (tumour induction) groups. Mice assigned to 4T1 groups were treated as follows:

Mice were anesthetized by inhalation of a mixture of air and 5% sevofluorane. After low abdomen asepsis, 4^th^ nipple was located and 10^4^ 4T1 cells were introduced by a single injection into the mammary fat pad. Tumour growth was monitored for 25 days.

### Flow cytometry

The spleen was mechanically disaggregated using a 50 µm nylon mesh and washed with PBS. Spleen erythrocytes were lysed with ACK buffer (150 mM NH_4_Cl, 10 mM KHCO_3_, 0.1 mM Na_2_EDTA, pH 7.3) for 10 minutes and washed with PBS. Tumour samples were finely cut and incubated 20 minutes in digestion medium (RPMI 1640, 10 U/ml DNase (Roche, Mannheim Germany), 0.5 mg/ml type IV Collagenase (Sigma, St. Louis MO)). Digestion was stopped by adding 50 µl FBS and mesh disaggregation was performed, followed by PBS wash. Cells from all tissues were resuspended in FACS buffer (PBS, 2% FBS, 0.02% NaN_3_).

Approximately 1 × 10^6^ cells were incubated with anti-CD16/CD23 (TruStain^®^, BioLegend, San Diego CA) for 30 minutes at 4 °C, washed and stained. For the characterization of cellular subpopulations, the following antibodies were used: APCCy7-conjugated anti- CD3ε (145-2C11), PE-conjugated anti-CD4 (GK1.5), AlexaFluor^®^647-conjugated anti-Foxp3 (150D), PerCP-conjugated anti-CD8 (53–6.7), AlexaFluor^®^647-conjugated anti-F4/80 (BM8), PE-conjugated anti-NKp46 (29A1.4) (all from BioLegend, San Diego CA), and VioletFluor^®^450-conjugated anti-CD25 (PC61.5) (Tonbo biosciences, San Diego CA). For intra-nuclear staining, Foxp3/Transcription Factor Staining Buffer kit (Tonbo biosciences, San Diego CA) was used according to manufacturer’s protocol. For oestrogen receptor alpha detection, a rabbit polyclonal anti-ERα (H-184) (Santa Cruz bt., Dallas TX) was used, followed by DyLight^®^488-conjugated Donkey anti-Rabbit IgG (BioLegend, San Diego CA). Using an Attune cytometer (Life Technologies) with blue and red laser, the obtained data was analysed with the FlowJo software (Treestar Inc.).

### Immunofluorescence

Tumour tissue samples were fixed in 4% paraformaldehyde (in PBS pH 7.1) for 20 minutes, cryoprotected in 30% sucrose (in PBS, pH 7.1) overnight, embedded in O.C.T. compound (Sakura Finetek, Torrance CA) and frozen at −70 °C. 10 µm thick sections were washed with PBS, permeabilised with 0.1% Triton X-100 (Sigma, St. Louis MO) in PBS for 10 min and blocked with 1% Bovine Serum Albumin (Sigma, St. Louis MO) for 2 hours. The sections were then incubated overnight at 4 °C with PE-conjugated anti-CD4 (GK1.5) and AlexaFluor^®^647-conjugated anti-Foxp3 (150D) (BioLegend, San Diego CA). After thoroughly washing with PBS, the sections were mounted in fluorescence mounting medium Fluoroshield^®^ (Sigma, St. Louis MO) and stored at 4 °C until examination with confocal microscope (LSM5 Pascal, Carl Zeiss).

### RT-PCR

Tumour tissue samples were frozen in TRIzol^®^ reagent (Ambion, Carlsbad CA) immediately after recollection. Total RNA was extracted with same reagent, following manufacturer’s protocol. Briefly, the tissue was disrupted in TRIzol^®^ reagent (1 ml/0.1 g tissue) and 0.2 ml of chloroform was added per each ml of reagent. After centrifugation at 13,000 rpm for 15 minutes, the aqueous phase was recovered. RNA was precipitated with isopropyl alcohol, washed with 75% ethanol, and dissolved in RNAse-free water. RNA concentration was determined by absorbance at 260 nm, and its integrity was verified following electrophoresis on 1.0% agarose gel.

Total RNA samples were immediately reverse-transcribed, using M-MLV Reverse Transcriptase (Promega, Madison WI) and dT12–18 primers (Invitrogen, USA). Then, cDNA was specifically amplified by semi-quantitative PCR, using TaqDNA polymerase (Biotecnologías Universitarias, UNAM. México) and *Mus musculus*-specific primers (see Table 1). The relative expression of each amplified gene was obtained by densitometric analysis, using the 18S-ribosomal RNA amplicon as a constitutive control.

**Table 1:** Oligonucleotide sequences used for RT-PCR.

Oligonucleotide	Sequence	MT (°C)	Product (bp)
IL-1β Sense	TCATGGGATGATGATGATAACCTGCT	62	502
IL-1β Antisense	CCCATACTTTAGGAAGACACGGATT		
IL-10 Sense	AACTGGTAGAAGTGATGCCCCAGGCA	63	237
IL-10 Antisense	CTATGCAGTTGATGAAGATGTCAAA		
TNF-α Sense	GGCAGGTCTACTTTGGAGTCATTGC	63	300
TNF-α Antisense	ACATTCGAGGCTCCAGTGAATTCGG		
IFN-γ Sense	AGCGGCTGACTGAACTCAGATTGTAG	60	247
IFN-γ Antisense	GTCACAGTTTTCAGCTGTATAGGG		
TGF-β Sense	CTTCAGCTCCACAGAGAAGAACTGA	61	298
TGF-β Antisense	CACAATCATGTTGGACAACTGCTCC		
18S Sense	CGCGGTTCTATTTTGTTGGT	60	219
18S Antisense	AGTCGGCATCGTTTATGGTC		
Arg-1 Sense	CTTGCGAGACGTAGACCCTG	64	387
Arg-1 Antisense	TGAGTTCCGAAGCAAGCCAA		
Fizz-1 Sense	GGTCCCAGTGCATATGGATGAGAC	58	296
Fizz-1 Antisense	CACCTCTTCACTCGAGGGACAGTT		
YM-1 Sense	TCACAGGTCTGGCAATTCTTCTG	60	437
YM-1 Antisense	TTTGTCCTTAGGAGGGCTTCCTC		
iNOS Sense	CAGCTCCACAAGCTGGCTCG	63	657
iNOS Antisense	CAGGATGTCCTGAACGTAGACCTT		

### Statistical analysis

The general experimental design considers 2 independent variables: neonatal exposure (Intact, Vehicle of BPA) and mammary tumour induction (Control or 4T1). The data regarding tumour development and tumour microenvironment only considers the exposure variable, as all animals belong to 4T1 group. Data from 2-3 independent experiments are charted as mean ± standard deviation and analysed with Prism 6^®^ software (GraphPad Software Inc.). Data distribution normality was assessed *via* Shapiro-Wilk test. Thereafter, a one-way ANOVA (α = 0.05) was performed, followed by a Tukey *post-hoc* test. Differences were considered significant when *p* < 0.05, with the actual *p* value being stated in each figure legend. The data regarding oestrogen receptor expression considers both independent variables and therefore, a two-way ANOVA (α = 0.05) was performed, followed by a Holm-Šidák *post-hoc* test, with the same significant difference criterion.

### Data availability

The datasets generated and analysed during the current study are available from the corresponding author on reasonable request.
